# *N*-Pyrrylarylsulfones with High Therapeutic Potential

**DOI:** 10.3390/molecules22030434

**Published:** 2017-03-09

**Authors:** Valeria Famiglini, Sabrina Castellano, Romano Silvestri

**Affiliations:** 1 Department of Drug Chemistry and Technologies, Sapienza University of Rome, Laboratory affiliated to Istituto Pasteur Italia—Fondazione Cenci Bolognetti, Piazzale Aldo Moro 5, I-00185 Roma, Italy; valeria.famiglini@uniroma1.it; 2 Department of Pharmacy, University of Salerno, Via Giovanni Paolo II 132, I-84084 Fiscano, Salerno, Italy; scastellano@unisa.it

**Keywords:** sulfonamide, heterocycle, polycyclic compound, therapeutic agent

## Abstract

This review illustrates the various studies made to investigate the activity of *N*-pyrrylarylsulfone containing compounds as potential antiviral, anticancer and SNC drugs. A number of synthetic approaches to obtain tetracyclic, tricyclic and non-cyclic compounds, and their biological activity with regard to structure–activity relationships (SARs) have been reviewed. The literature reviewed here may provide useful information on the potential of *N*-pyrrylarylsulfone pharmacophore as well as suggest concepts for the design and synthesis of new *N*-pyrrylarylsulfone based agents.

## 1. Introduction

**Chart 1:**
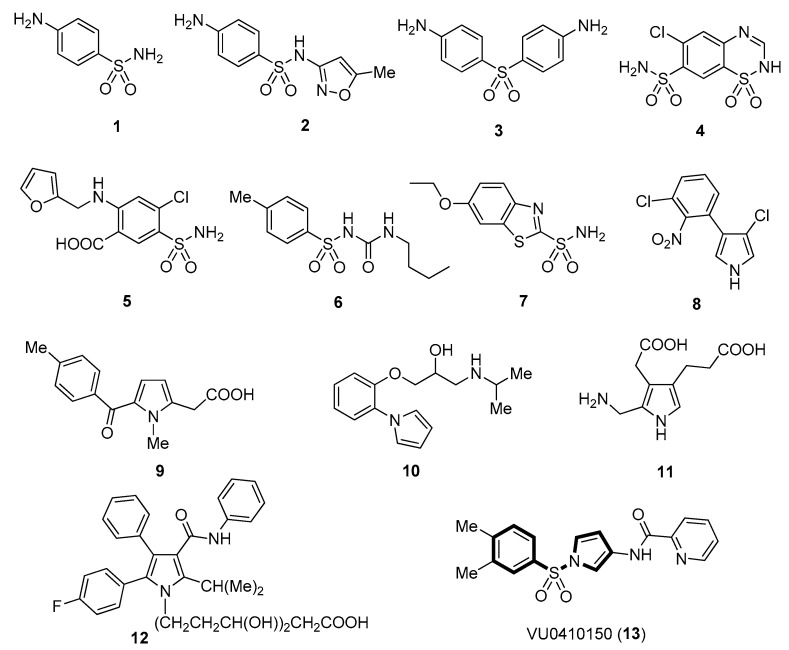
Examples of sulfa and pyrrole containing drugs.

Sulfonamide is the basis of several groups of drugs [1]. Intense interest focused on sulfonamide drugs after the discovery in 1935 that the activity of red dye Prontosil [2,3] was attributed to breakdown product sulfanilamide (**1**). The antibacterial sulfonamides work as competitive inhibitors of the dihydropteroate synthase, an enzyme involved in folate synthesis [4]. The simple **1** was cheaper than Prontosil, had fewer unwanted effects and did not impart the typical red color to the skin. Nowadays, sulfonamides have been replaced by other antibacterial drugs such as β-lactam antibiotics, with some important exception; for example, sulfamethoxazole (**2**) is used for treatment of urinary and respiratory-tracts infections [5]. Sulfa molecules have been chemically manipulated to obtain drug for the treatment of leprosy, fluid accumulation and diabetes. The modern era of drug treatment of leprosy began in 1937 when the sulfa drug dapsone (**3**) [6] proved to be highly effective. For more than six decades, **3** remained first line drug to treat leprosy. Since 1980s, **3** has been administered in combination with rifampicin and clofazimine for treatment of leprosy [7]. Chlorothiazide (**4**) is a carbonic anhydrase inhibitor which was introduced in 1958 as a diuretic drug and is used to treat hypertension and edema [8,9]. Before the discovery of **4**, mercurial drugs associated with severe toxicity were the only available drugs to treat fluid retention. Few years later, in 1962, another sulfonamide, furosemide (**5**), was discovered as diuretic drug and is used to treat fluid retention and for the treatment of high blood pressure [10]. Tolbutamide (**6**), the first sulfonylurea anti-diabetic drug, was approved in the United States in 1957 for the treatment of type 2 diabetics [11]. Even though since 1964 there were concerns that sulfonylurea antidiabetic drugs may increase cardiovascular risk, the current literature does not confirm the detrimental risk profile of sulphonylureas compared with other anti-diabetic drugs [12]. Ethoxyzolamide (**7**) is a carbonic anhydrase inhibitor used in the treatment of glaucoma and duodenal ulcers, and as a diuretic [13]. Other sulfa drug examples include antivirals agents, such HIV-1 non-nucleoside reverse transcriptase and protease inhibitors [14,15,16,17,18], HCV NS3/4A protease [19] and NS5B polymerase inhibitors [20]; antibiotics, such mafenide, approved by the FDA in 1948 [21]; and nonsteroidal anti-inflammatory drug such celecoxib, a COX-2 selective inhibitor [22] (Chart 1).

Pyrrole ring is a well-known privileged scaffold that exhibits a wide variety of biological activities [23]. Including the pyrrole into different pharmacophores has resulted in non-cyclic and polycyclic pyrrole-containing systems with potential therapeutic effects such as anticancer (leukemia and lymphoma), anti-microbic (bacteria, malaria, protozoa, and fungi) and central nervous system agents (antipsychotic and anxiolytic) (for example, pyrrolnitrin (**8**) [24], tolmetin (**9**) [25], isamoltane (CGP-361A) (**10**) [26], porphobilinogen (**11**) [27], and atorvastatin (**12**) [28]). Recently, VU0410150 (**13**), a pyrrylarylsulfone containing compound, has been discovered as mGluR4-positive allosteric modulator and evaluated as potential drug for treatment for Parkinson’s disease [29,30]. In the past decades, numerous *N*-pyrrylarylsulfones have been synthesized by our research group in several drug discovery projects. In this work, attempt has been made to review various *N*-pyrrylarylsulfone based compounds to discuss the synthetic approaches and the biological activity with regard to structure–activity relationships (SARs).

## 2. Pyrrolo[1,2-*b*]-*s*-triazolo[3,4-*d*][1,2,5]benzothiadiazepine 5,5-dioxide

**Scheme 1:**
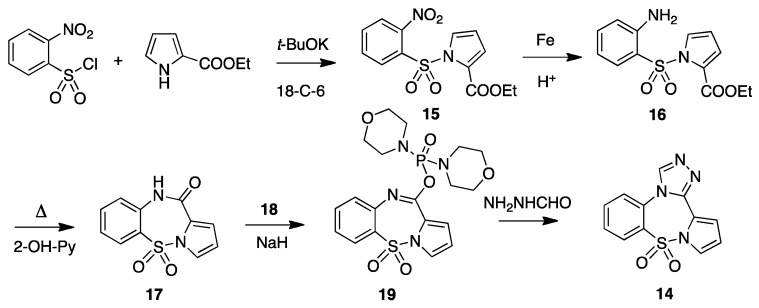
Synthesis of **14**.

Tetracyclic systems, for example mianserin, aptazepine and bretazenil, have been widely investigated as psychotic drugs. The synthesis of pyrrolobenzothiadiazepine anellated with azole ring started as a development of a previous research project on tetra-anellated heterocycles [31,32,33]. Pyrrolo[1,2-*b*]-*s*-triazolo[3,4-*d*][1,2,5]benzothiadiazepine 5,5-dioxide (**14**) was synthesized by reaction of 2-nitrobenzensulfonyl chloride with ethyl pyrrole-2-carboxylate in the presence of potassium *tert*-butoxide and 18-crown-6 to provide 2-ethoxycarbonyl-1-(2-nitrobenzenesulfony)-1*H*-pyrrole (**15**). After reduction of **15** to amino derivative **16**, the product was cyclized to lactam **17** in the presence of 2-hydroxypyridine as a bifunctional catalyst. Treatment of **17** with di-4-morpholinylphosphinic chloride (**18**) in the presence of sodium hydride afforded phosphinyloxyimine **19** which was transformed into **14** by reaction with formylhydrazine (Scheme 1) [34].

## 3. 2-Methyl-1,3,4,14b-tetrahydro-2*H*-pyrazino[2,1-*d*]pyrrolo[1,2-*b*][1,2,5]benzothiadia-zepine 10,10-dioxide

**Scheme 2:**
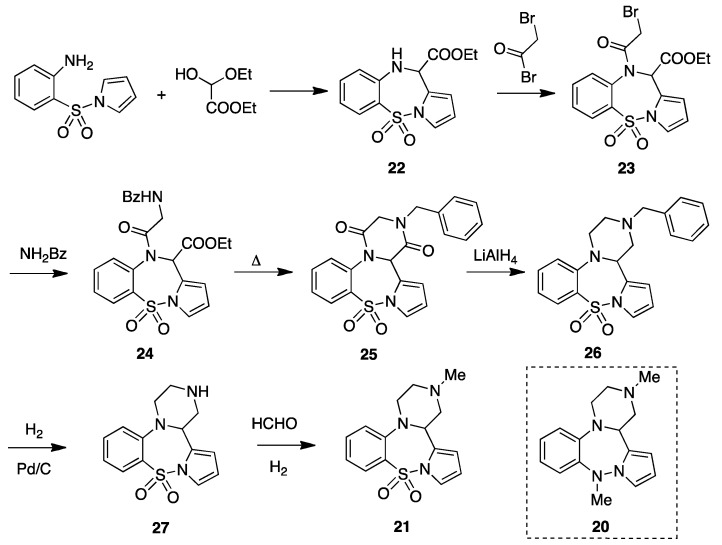
Synthesis of **21**, and structure of 10-methyl-10-azaaptazepine (**20**).

Studies on tetracyclic analogs of mianserin as antidepressant drugs led to the development of the pyrrole analog aptazepine and the strictly related isoaptazepine and 10-methyl-10-azaaptazepine (**20**). Pursuing this research project, 2-methyl-1,3,4,14b-tetrahydro-2*H*-pyrazino[2,1-*d*]pyrrolo[1,2-*b*][1,2,5]benzothiadiazepine 10,10-dioxide (**21**) (tiaaptazepine) was designed as new putative core for central nervous system (CNS) active drugs. The synthesis of **21** is depicted in Scheme 2. Reaction of 1-(2-aminobenzenesulfonyl)pyrrole with ethyl glyoxylate via a Pictet-Spengler type condensation gave 11-ethoxycarbonyl-10,11-dihydropyrrolo[1,2-*b*][1,2,5]benzothiadiazepine 5,5-dioxide (**22**). Reaction of **22** with bromoacetyl bromide afforded the corresponding bromoacetyl derivative **23** which reacted with benzylamine (**24**) and subsequently thermally cycled to **25**. Compound **25** was reduced with lithium aluminum hydride (**26**) and debenzylated to **27** with hydrogen over Pd/C. Finally, **27** was converted to **21** via reductive amination using formaldehyde in the presence of hydrogen (Scheme 2) [35].

**Scheme 3:**
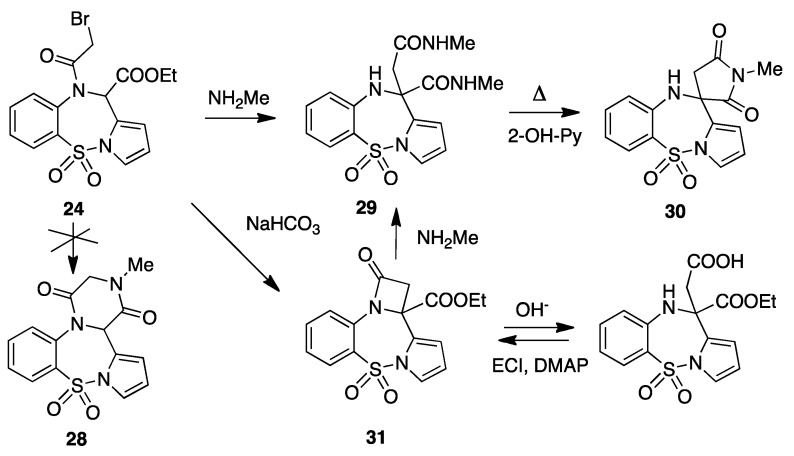
Chemical transformation of **24**.

It is worthwhile mentioning that direct cyclization of **24** in the presence of excess of methylamine via diketo intermediate **28**, failed due the formation 11-carboxy-10,11-dihydropyrrolo[1,2-*b*][1,2,5]benzothiadiazepine-11-acetic acid bis methylamide 5,5-dioxide (**29**). Intramolecular cyclization of **29** in the presence of 2-hydroxypyridine led exclusively to the spiro derivative **30**. It should be noted that treatment of **24** with sodium hydrogen carbonate gave lactam **31**, which might be the intermediate of the conversion of **24** to **29** (Scheme 3) [36].

Compounds **21** and **22** were enantioseparated by enantioselective HPLC, and the absolute configuration of the pure enantiomers was established by circular dichroism (CD) spectroscopy.

The in vitro binding affinities for several CNS receptors (DA_1_, DA_2_, DA_3_, 5-HT_1A_, 5-HT_2A_, 5-HT_2C_, 5-HT_3_, α_1NA_, α_2NA_ and muscarinic receptors) showed that both enantiomers of derivative **21**, (−)-(*R*)-**21** and (+)-(*S*)-**21**, showed higher affinities than the (−)-(*R*)-**22** and (+)-(*S*)-**22** counterparts, with exception of α_1NA_ for which (+)-(*S*)-**22** was superior. Compound (+)-(*S*)-**21** showed good affinities for 5-HT_1A_, 5-HT_2A_, 5-HT_2C_, and α_1NA_ receptors but only moderate affinities for DA_1_, DA_2_ and 5-HT3 receptors. Compared to the reference compounds mirtazepine, mianserine and 5-methoxymianserin, this compound showed higher affinity of the 5-HT_1A_ subtype, and different general pharmacological profile [37].

## 4. Imidazo[5,1-*d*]pyrrolo[1,2-*b*][1,2,5]benzothiadiazepine 9,9-dioxide

**Scheme 4:**
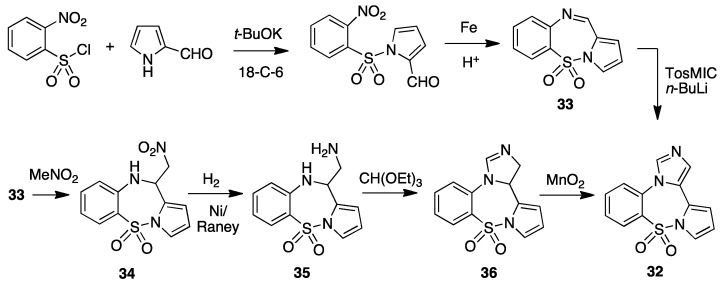
Synthesis of **32**.

Imidazo[5,1-*d*]pyrrolo[1,2-*b*][1,2,5]benzothia-diazepine 9,9-dioxide (**32**) was synthesized as a new benzothiadiazepine tetracyclic ring of pharmaceutical interest. The synthesis of **32** was achieved by a simple procedure involving the anellation of pyrrolo[1,2-*b*][1,2,5]benzothiadiazepine 5,5-dioxide (**33**) at the 10,11-azomethine bond by cycloaddition with tosylmethyl isocyanide (TosMIC) in the presence of buthyl lithium. Alternatively, **32** could be prepared starting from addition reaction of nitromethane to the azomethine bond of **33** to provide **34** which was reduced to amino **35** with of hydrogen at high pressure in the presence of nickel/Raney as a catalyst. Treatment of **35** with triethyl orthoformate furnished the dihydro derivative **36** which was oxidized to **32** with manganese dioxide (Scheme 4) [38].

## 5. 5*H*-pyrrolo[1,2-*b*][1,2,5]benzothiadiazepin-11(10*H*)-one 5,5-dioxide

5*H*-pyrrolo[1,2-*b*][1,2,5]benzothiadiazepin-11(10*H*)-one 5,5-dioxide (PBTD) derivatives, analogs of compound **17** described in Scheme 1, were synthesized as a novel class of HIV-1-specific non-nucleoside reverse transcriptase inhibitors (NNRTIs). In general, the newly synthesized compounds were non cytotoxic for MT-4 cells at concentrations up to 300 μM. Maximum antiviral activity was obtained with compounds **37a**–**h** bearing the chlorine atom at position 7 and the alkyl/alkenyl group at position 10 of the pyrrolo[1,2-*b*][1,2,5]benzothiadiazepine ring (Table 1). Compounds **37a** and **37b** (EC_50_ = 1.0 and 0.5 μM, respectively) showed the highest potency and selectivity (SI of >300 and >600, respectively) [39].

Crystal structure [40] of **37a** showed that the aromatic moieties adopted a dihedral angle of 114.4°, a value that was very near to the optimal value of the butterfly-like conformation reported by the Schaefer’s model [41].

## 6. Pyrrolo[1,2-*b*][1,2,5]benzothiadiazepine Acetic Acid 5,5,-dioxide

**Scheme 5:**
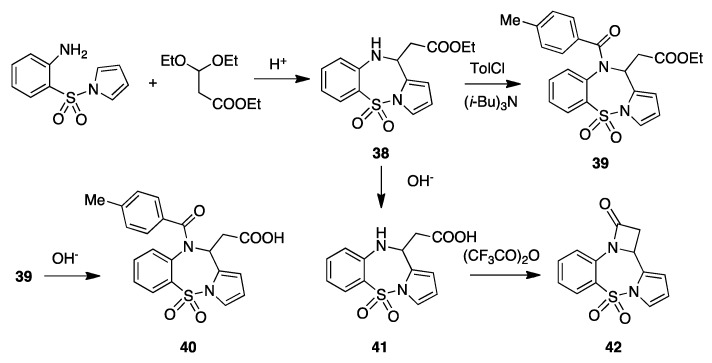
Synthesis of **38** and **42**.

The PBTD scaffold has been exploited in several antiviral research programs. A series of pyrrolo[1,2-*b*][1,2,5]benzothiadiazepine acetic acid derivatives was synthesized by reaction of 1-(2-aminobenzenesulfonyl)pyrrole with ethyl 3,3-diethoxypropionate in aqueous acetic acid to furnish ethyl 10,11-dihydro-pyrrolo[1,2-*b*][1,2,5]benzothiadiazepine-11-acetic acetate 5,5-dioxide (**38**). Ester **38** was *N*-acylated in the presence of triisobutylamine to afford ethyl 10,11-dihydro-10-(4-methylbenzoyl)pyrrolo[1,2-*b*][1,2,5]benzothiadiazepine-11-acetate 5,5-dioxide (**39**) which was hydrolyzed into the corresponding acetic acid **40**. Alternatively, alkaline hydrolysis of **38** furnished acid **41** which was transformed into azetidone **42** by treatment with trifluoroacetic anhydride (Scheme 5). Derivatives **38** and **42** showed significant inhibition of HIV-1 with EC_50_ = 19.5 and 18 μM, respectively) [42].

**Chart 2:**
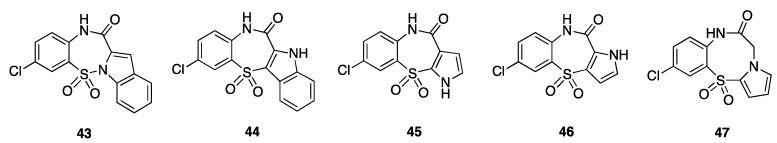
Heterocyclic compounds structurally correlated to PBTD HIV-1 NNRTIs.

Replacement of the pyrrole ring of PBTD with the indole (**43**) resulted in weaker antiretroviral compounds [39]. On the other hand, the 5*H*-indolo[3,2-*b*][1,5]benzothiazepine isomers (e.g., **44**), were endowed with anti-HIV-1 activity in the low micromolar range of concentrations [43]. In addition, 1*H*-pyrrolo[2,3-*b*][1,5]benzothiazepine (e.g., **45**), 1*H*-pyrrolo[3,2-*b*][1,5]benzothiazepine (e.g., **46**) [44] and 9*H*-pyrrolo[2,1-*b*][1,3,6]benzothiadiazocin-10(11*H*)-one 4,4-dioxide derivatives (**47**) [45] were synthesized as new heterocyclic systems mimicking the structural features of the PBTDs scaffold (Chart 2).

## 7. PBTDs as Chronic Myelogenous Leukemia (CML) Agents

**Chart 3:**
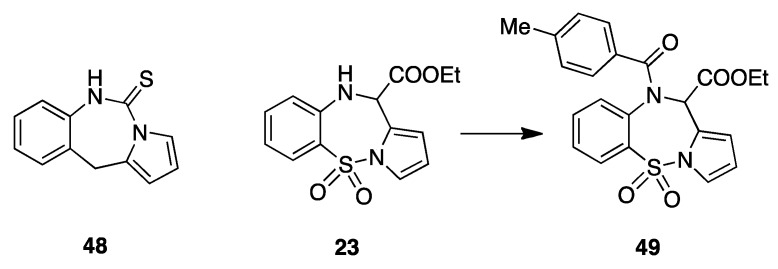
PBTDs as CML agents.

The antitumor activity of pyrrolo[2,1-*c*][1,4]benzodiazepines (PBDs, e.g., **48**) related to anthramycin was extensively studied, as it was documented in Thurston’s review [46]. Given the high structural similarity between PBTD and PBD compounds, two PBTDs, **23** and its 10-(4-methylbenzoyl) derivative **49**, were selected for screening of pro-apoptotic and anti-leukemia activity (Chart 3, Table 2 and Table 3) [47]. PBTD **23** was prepared by an improved procedure using dimethoxyacetal of ethyl glyoxylate in absolute ethanol in the presence of 4-toluenesulfonic acid (PTSA). Compound **23**, prepared as described in Scheme 2, was *N*-acylated to **49** with 4-methylbenzoyl by refluxing in 1-bromo-3-chloropropane in the presence of sodium hydrogen carbonate. PBTDs **23** and **49** induced apoptosis in K562 cells and caused cell death in BCR-ABL-positive leukemia cells obtained from chronic myeloid leukemia patients who were at onset or were IM-resistant. Apoptotic mechanism studies showed that PBTDs **23** and **49** activated the caspase activity through two different pathways: both compounds activated caspase-3; **23** significantly reduced the procaspase-8; in contrast **49** evidenced a decrease of procaspase-9 band. The apoptosis was observed before the expression of BCR-ABL protein and the tyrosine phosphorylation. PBTDs-mediated suppression of K562 cell proliferation was characterized by the appearance of DNA fragmentation and was associated with the poly(ADPribose) polymerase (PARP) cleavage. PBTDs **23** and **49** treatment resulted in caspase-3 activation through down-regulation of Bcl-2 and up-regulation of Bax [48]. PBTDs possessed inhibitory activity against mTOR and impeded hyper-phosphorylation of Akt as a feedback of inhibition of mTOR by rapamycin [49]. These findings highlighted PBTDs as potential agents for the treatment of CML [50,51].

## 8. Pyrryl Aryl Sulfones

**Scheme 6:**
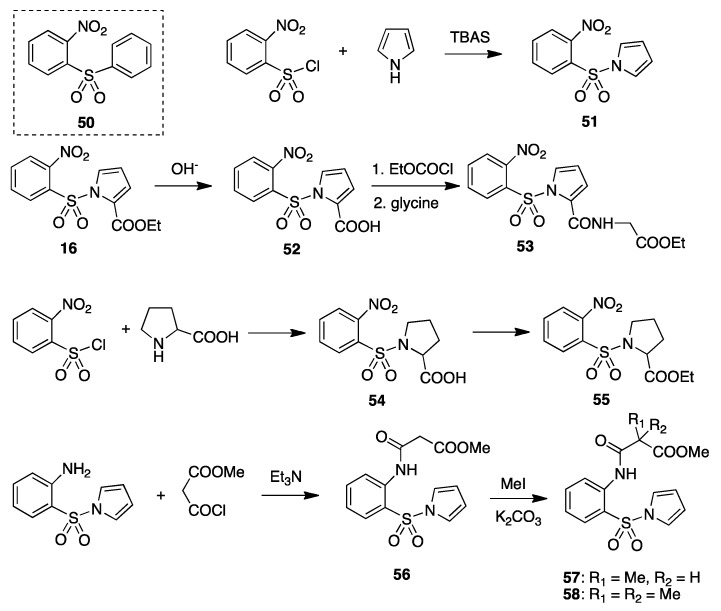
Synthesis of PAS **51**–**58**.

Diarylsulfones emerged as a chemical class of HIV-1 NNRTIs. The presence of the nitro group at position 2 of the phenyl ring and the sulfur bridging atom as sulfur dioxide are fundamental structural characteristics for their activity. The antiviral activity of 2-nitrophenyl phenyl sulfone (**50**, NPPS) [52] prompted the synthesis of a series of 41 pyrryl aryl sulfones (PAS) and some related derivatives [53]. Pyrryl 2-nitrophenyl sulfone (**51**) was straightforwardly prepared by nucleophilic substitution reaction between 2-nitrobenzenesulfonyl chloride and pyrrole in the presence of *n*-tetrabutylammonium hydrogen sulfate (TBAS) as a phase transfer catalyst. On the other hand, alkaline hydrolysis of 2-ethoxycarbonylpyrrole (**16**) [34] afforded the acid **52** which was transformed into **53** by reaction with ethyl chloroformate in the presence of 4-methylmorpholine followed by treatment of the intermediate mixed anhydride with glycine ethyl ester (Scheme 6).

Ester **55** was prepared by treating the corresponding acid **54** [54] with oxalyl chloride and then with anhydrous ethanol. Reaction of 1-(2-aminobenzenesulfonyl)pyrrole [35] with methyl malonyl chloride in the presence of triethylamine led to amide **56** which in turn was methylated to **57** or **58** with one or two equivalents of methyl chloride, respectively, in the presence of potassium carbonate (Scheme 6). Compound **16**, a 2-nitrophenyl 1-pyrryl sulfone bearing the 2-ethoxycarbonyl function, showed the highest anti HIV-1 activity (Table 4).

The importance of the diaryl sulfone moiety for the design of new anti-HIV-1 agents was further confirmed by the synthesis of new series of PAS and indolyl aryl sulfones [55,56]. The amino-PAS derivatives were synthesized as follows. Alkylation of the 2-amino group was achieved by reaction of **59a** and **59b** with the appropriate aldehyde in the presence of sodium cyanoborohydride; carboxamides were obtained by heating with an acyl chloride in pyridine (Scheme not shown). It was reported that the 4-chloroaniline moiety or the related 5-chloro-2-pyridylamine represented the key feature of highly potent HIV-1 NNRTIs, for example 8-Cl-TIBO [57], 7-Cl-PBTD (**37**) [39] (Table 1), 3,3-dialkyl-3,4-dihydroquinoxaline-2-(1*H*)thione [58], oxoquinoline [59], and PETT [60]. In the case of PAS derivatives, the 4-chloroaniline moiety worked as a pharmacophore only when the sulfonyl group was near to the amino group. The nature of the pharmacophore could not be modified without affecting the anti-HIV-1 activity. The highest anti-HIV-1 activity of compounds **59a** and **59b** was also associated with the presence of the alkoxycarbonyl group at position 2 of the pyrrole ring. Alkylation of aniline nitrogen completely abolished the activity (data not shown), whereas acylation led to weakly active compounds (Table 5). The ability to inhibit the recombinant reverse transcriptase (rRT) of HIV-1 is depicted in Table 6. When tested against the rRT form HIV-1 mutants resistant to nevirapine (Y181C) and TIBO (L1001I), the compounds showed activity at 10-fold higher concentrations.

Compound **59b** was selected as lead compound for an antiviral project based on molecular modeling studies. Using the three-dimensional structure of HIV-1 RT cocrystallized with α-APA (alpha-anilinophenyl acetamide) derivative R95845, a model of RT/**59b** complex was derived using previously developed SARs. The experimentally determined RT bound conformations of α-APA R90385 [61] served as basis to select conformations of **59b** for docking studies. By scanning the rotatable bonds of the crystal structure of **59b**, a low energy conformation was identified and this compound superimposable on α-APA R95845 about the aromatic rings and the COOEt/COMe and SO_2_/CONH_2_ groups. Inspection of the RT/**59b** complex revealed a region of the HIV-1 NNBS (non-nucleoside binding site) delimited by Tyr181, Tyr188 and Trp229 side chains, which could be filed by substituents at position 4 of the pyrrole ring. Among the compounds synthesized, **60** (EC_50_ = 42 nM; IC_50_ = 50 nM) was the most potent PAS derivative (Table 7). Compared with **59b**, it showed three- and eight-fold improvement in cell-based and enzyme assays, respectively [62].

Further studies were conducted to improve the activity of PAS **59b** [63]. New PAS derivatives were synthesized by introduction of different alkyl, alkenyl or cycloalkyl substituents at the 2-ester function, along with a small series of 2-carboxamide derivatives, in order to explore the effects of substituents a position of the pyrrole ring. The new derivatives were less potent and sometimes more toxic than the previously reported **59a** and **59b**. This study confirmed the key role of the 4-chloroanilino moiety and the substituent at the ester function.

**Scheme 7:**
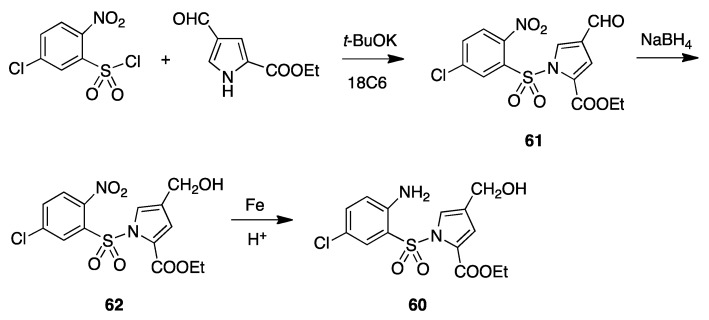
Synthesis of PAS **60**.

Compound **60** was synthesized as depicted in Scheme 7. 5-Chloro-2-nitrobenzenesulfonyl chloride was reacted with 2-ethoxycarbonyl-1*H*-pyrrole-4-carboxaldehyde in the presence of potassium *tert*-butoxide and 18-crown-6 to give **61**. Sodium borohydride reduction of aldehyde **61** afforded alcohol **62**, and the nitro group reduction with iron in glacial acetic acid to provide the amino derivative **60** (Scheme 7).

**Chart 4:**
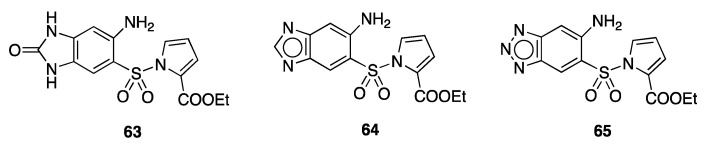
Structure of pyrryl heteroaryl sulfones **63**–**65**.

Three pyrryl heteroaryl sulfones, ethyl 1-[(6-amino-2-oxo-2,3-dihydro-1*H*-benzo[*d*]imidazol-5-yl)sulfonyl]-1*H*-pyrrole-2-carboxylate (**63**), ethyl 1-[(5-amino-1*H*-benzo[*d*]imidazol-6-yl)sulfonyl]-1*H*-pyrrole-2-carboxylate (**64**), and ethyl 1-[(6-amino-2*H*-benzo[*d*][1,2,3]triazol-5-yl)sulfonyl]-1*H*-pyrrole-2-carboxylate (**65**), were designed as novel HIV-1 NNRTIs using structure based computational methods (Chart 4) [64]. These compounds inhibited the HIV-1 RT at micromolar concentrations, but were found inactive in the MT-4 cells assay.

## 9. Acylamino Pyrryl Aryl Sulfones

**Chart 5:**
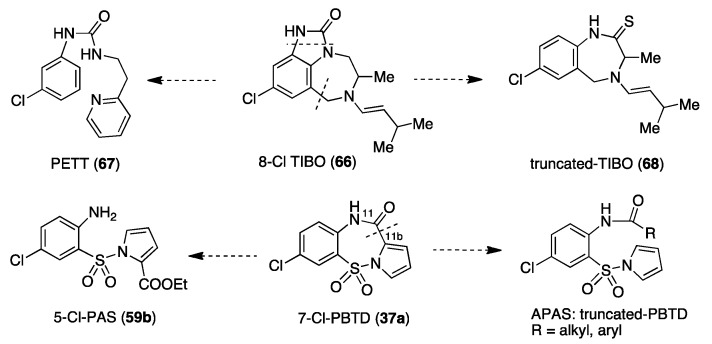
Design of APAS derivatives.

A series of PAS related compounds bearing acylamino moieties at position 2 of the benzene ring were synthesized as truncated analogs of PBTDs [39]. Furthermore, potent HIV-1 NNRTIs, such as PETT (**67**) [65] and truncated-TIBO (**68**) [66] compounds, were designed and synthesized based on the structure of 8-Cl-TIBO (**66**) [67,68] using a ring-opening strategy. Based on these findings, the same strategy was applied to 7-Cl-PBTD (**37a**) by breaking the 11,11b bond. The drug design strategy conceived a series of acylamino-PAS (APAS) derivatives, which were synthetized and characterized for their antiviral properties [69] (Chart 5).

Several APAS derivatives inhibited the HIV-1 replication in MT-4 cells in the 1–2 μM range. Two compounds, **69** and **70**, showed activity at submicromolar concentrations with EC_50_ of 0.4 and 0.5 μM, respectively. Both compounds failed to inhibit the HIV-1 K103N and Y181C mutant strains, similar to that observed for structurally correlated 2-amino-6-[(3,5-dimethyl)sulfonylbenzonitrile [70]. Although structurally related to the previously reported PAS family, the APAS derivatives were investigated for binding mode in the non-nucleoside binding site of the HIV-1 RT [71]. Derivative **69**, the most active among the test APASs, was modeled from the X-ray coordinates of **59b** and docked into the HIV-1 NNBS of the RT using the 2-amino-6-[(3,5-dimethyl)sulfonylbenzonitrile/RT complex [70]. The binding mode of **69** shared similarities with previously reported PASs [62,64]: the ethoxycarbonyl filled the highly hydrophobic region of NNBS, and the 4-chloro-2-methoxycarbonyl moiety took up the H-bond region.

**Scheme 8:**
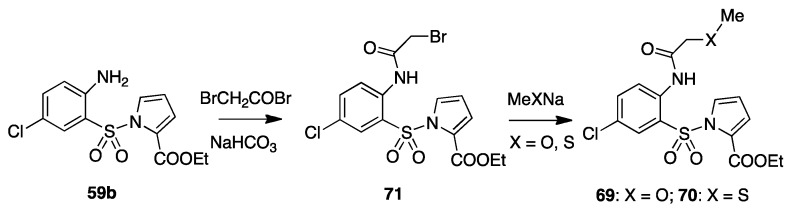
Synthesis of APASs **69** and **70**.

APAS derivatives **69** and **70** were prepared by reacting compound **59b** with bromoacetyl bromide 1-bromo-3-chloropropane in the presence sodium hydrogen carbonate to give 2-bromoacetylamino derivative **71**. Treatment of **71** with sodium methoxide or thiomethoxide afforded APASs **69** or **70**, respectively (Scheme 8).

## 10. Smiles Rearrangement

**Scheme 9:**
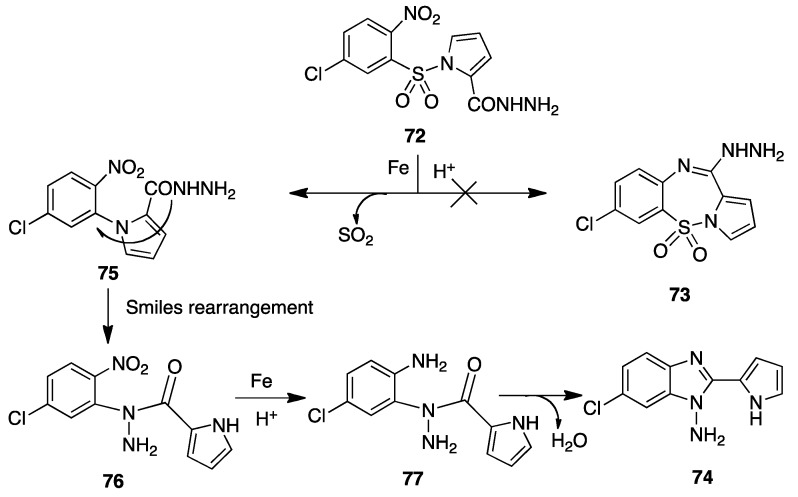
Smile rearrangement of **72** to **74**.

In the search for novel tetracyclic ring systems containing the benzothiadiazepine ring, a multistep synthesis was planned starting from 1-[(5-chloro-2-nitrophenyl)sulfonyl]-1*H*-pyrrole-2-carbohydrazide (**72**) [72]. Reduction of **72** with iron powder in glacial acetic acid did not afford the expected 7-chloro-11-hydrazinopyrrolo[1,2-*b*][1,2,5]benzothiadiazepine (**73**), but only a bicyclic derivative that was identified as 1-amino-6-chloro-(1*H*-pyrrol-yl)benzimidazole (**74**). Structure of **74** was established by NMR spectroscopy and elemental analysis, and was confirmed by crystallographic data. Formation of **74** was hypothesized by extrusion of the sulfur dioxide followed by Smiles rearrangement [73] of **75** to **76**. Reduction of nitro group to amino underwent with concomitant cyclization of the intermediate amino derivative **77** to form **74** (Scheme 9). The structure of **74** was confirmed by direct synthesis of **75** and subsequent treatment with iron in acetic acid to provide **74**. It is interesting to note that any attempt to obtain **73** from 1-[(5-chloro-2-aminophenyl)sulfonyl]-1*H*-pyrrole-2-carbohydrazide (the corresponding amino derivative of **72**), by heating in the presence of 2-hydroxypyridine, failed, **37a** being the only product of reaction.

## 11. Structurally Related Compounds

**Chart 6:**
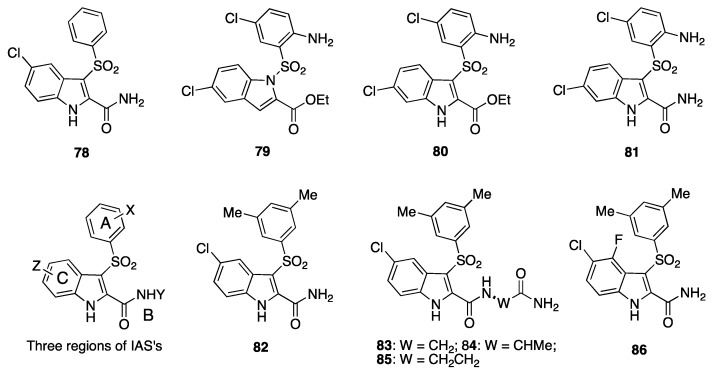
Structure of indolylsulfones **78**–**86**.

The highly potent anti-HIV-1 activity displayed by Merck carboxamide L-737,126 (**78**) [74,75,76] (HIV-1 WT_IIIB_ EC_50_ = 1 nM; HIV-1 RT IC_50_ = 25 nM) prompted the design of new indolylarylsulfone (IAS) analogs. Due to the lack of SAR information, the design of first IAS derivatives was based on PASs’ structural features. In general, 2-ethoxycarbonyl-1-benzenesulfonyl-1*H*-indoles showed weak antiretroviral activity, with the exception of derivative **79** (HIV-1 WT_IIIB_ EC_50_ = 8.3 μM) bearing the 4-chloroaniline moiety [77]. Indoles bearing the carbethoxy group at position 3 of the indole were inactive. Moving the 1-benzenesulfonyl group of **79** to position 3 of the indole gave IAS **80** (HIV-1 WT_IIIB_ EC_50_ = 1.9 μM) that showed 4.3-fold improvement of activity. Replacement of the 2-ester group with a carboxyamide function, **81** (HIV-1 WT_IIIB_ EC_50_ = 0.04 μM) led to a notably increase of both potency and selectivity. SAR studies led to partition the IAS scaffold in three regions: (A) the activity of **78** against HIV-1 mutant strains significantly improved by the presence of two methyl groups at positions 3 and 5 of the 3-phenylsulfonyl moiety (**82**) [78]; (B) coupling the indole-2-carboxamide with either natural or unnatural amino acids provided potent HIV-1 inhibitors, for example **83**–**85**, against the HIV-1 L100I, K103N, and Y181C strains in CEM cells, with potency comparable to the fist line HIV-1 NNRTI efavirenz [79,80]; and (C) the 5-chloro-4-fluoro substitution pattern at the indole ring, compound **86**, afforded potent inhibitors of HIV-1 RT WT and RTs carrying the K103N, Y181I, and L100I mutations [81] (Chart 6).

## 12. Conclusions

*N*-pyrrylarylsulfones display a variety of biological activities. This review illustrates the various studies made to investigate the *N*-pyrrylarylsulfone scaffold as privileged structure to discover putative antiviral, anticancer and SNC drugs. A number of synthetic approaches to obtain tetracyclic pyrrolo[1,2-*b*]-*s*-triazolo[3,4-*d*][1,2,5]benzothiadiazepine 5,5-dioxide, 2-methyl-1,3,4,14b-tetrahydro-2*H*-pyrazino[2,1-*d*]pyrrolo[1,2-*b*][1,2,5]-benzothiadiazepine 10,10-dioxide, imidazo[5,1-*d*]pyrrolo[1,2-*b*][1,2,5]benzothia-diazepine 9,9-dioxide, tricyclic 5*H*-pyrrolo[1,2-*b*][1,2,5]benzothiadiazepin-11(10*H*)-one 5,5-dioxide (PBTD), and non-cyclic pyrryl aryl sulfone and acylamino-PAS (APAS) compounds and their biological activity with regard to structure–activity relationships (SARs) have been reviewed. The literature reviewed here may provide useful information on the potential of *N*-pyrrylarylsulfone pharmacophore as well as suggest concepts for the design and synthesis of new *N*-pyrrylarylsulfone based agents.

## Figures and Tables

**Table 1:**
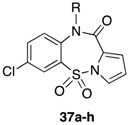
Anti-HIV-1 activity of 7-Cl-PBTDs **37a**–**h** ^a^.

Compound	R	HIV-1 III_B_		
		CC_50_^b^ (μM)	EC_50_^c^ (μM)	SI ^d^
**37a**	H	>300	1.0	>300
**37b**	Me	>300	0.5	>600
**37c**	Et	283	2.4	118
**37d**	Propyl	126	14	9
**37e**	Isopropyl	>300	Nd ^e^	-
**37f**	Allyl	>300	3.7	>81
**37g**	Crotyl	>300	4.1	>73
**37h**	Dimethylallyl	>300	129	>2

^a^ Data are mean values of two to three independent experiments each one in triplicate. ^b^ CC_50_: cytotoxic concentration (μM) to induce 50% death of noninfected cells, as evaluated with the MTT method in MT-4 cells. ^c^ EC_50_ (HIV-1, III_B_): effective concentration (μM) to inhibit by 50% HIV-1 (III_B_ strain) induced cell death, as evaluated with the MTT method in MT-4 cells. ^d^ SI: selectivity index calculated as CC_50_/EC_50_ ratio. ^e^ nd, no data.

**Table 2:** Apoptotic activity of **23** and **49** in cells from CML patients at onset at 10 μM [46].

Patient	Sex	Age	Source ^a^	% of Apoptosis			
				23		49	
				24 h	48 h	24 h	48 h
1	M	45	PB ^a^	64	70	77	85
2	M	60	BM ^b^	50	70	70	85
3	F	73	PB	65	79	66	82
4	M	83	BM	50	70	50	75
5	F	46	PB	50	70	60	80
6	F	27	PB	50	70	60	80
7	F	45	PB	60	80	64	80
8	M	35	PB	60	80	65	85
9	M	66	PB	60	80	60	80
10	F	38	PB	50	70	70	80
11	F	65	PB	52	71	55	78
12	F	27	PB	52	73	55	78

^a^ PB: peripheral blood cells. ^b^ BM: bone marrow cells.

**Table 3:** Apoptotic activity of **23** and **49** in cells from CML patients in blast crisis and Imatinib-resistant at onset at 10 μM.

Patient	Sex	Age	Source ^a^	Percent Apoptosis			
				23		49	
				24 h	48 h	24 h	48 h
13	M	38	PB ^a^	60	80	40	60
14	F	70	PB	55	78	50	70

^a^ PB: peripheral blood cells.

**Table 4:** Anti-HIV-1 Activity of PASs **16** and **51**–**58** ^a^.

Compound		HIV-1 III_B_	
	CC_50_^b^ (μM)	EC_50_^c^ (μM)	SI ^d^
**16**	>308	15.08	>20
**51**	36.55	>36.55	-
**52**	>337.5	>337.5	-
**53**	>262.2	>262.2	-
**54**	>333	>333	-
**55**	255	>255	-
**56**	>370	63	>5.8
**57**	>279	>279	-
**58**	>285	>285	-
**NPPS**	-	1.4	-

^a^ Data are mean values of two to three independent experiments each one in triplicate; ^b^ CC_50_: cytotoxic concentration (μM) to induce 50% death of non-infected cells, as evaluated with the MTT method in MT-4 cells; ^c^ EC_50_ (HIV-1, III_B_): effective concentration (μM) to inhibit by 50% HIV-1 (III_B_ strain) induced cell death, as evaluated with the MTT method in MT-4 cells; ^d^ SI: selectivity index calculated as CC_50_/EC_50_ ratio.

**Table 5:**
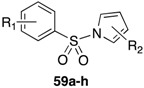
Anti-HIV-1 activity of amino-PAS **59a**–**h** against the WT strain ^a^.

Compound	R^1^	R^2^	HIV-1 III_B_		
			CC_50_^b^ (μM)	EC_50_^c^ (μM)	SI ^d^
**59a**	2-NH_2_-5-Cl	2-COOMe	>300	0.18	>2140
**59b**	2-NH_2_-5-Cl	2-COOEt	>300	0.14	>2140
**59c**	2-NO_2_	2-COOEt	>300	15	>20
**59d**	2-Cl	2-COOEt	141	25	5
**59e**	2-NH_2_-5-Cl	2-COOCH_2_CHC=CH_2_	100	0.40	250
**59f**	2-NHCHO-5-Cl	2-COOEt	>300	1.0	>300
**59g**	2-NHCOMe-5-Cl	2-COOEt	≥300	1.0	≥300
**59h**	2-NHCOOEt	2-COOEt	>300	1.0	>300
NVP ^e^			>10000	0.60	>167

^a^ Data are mean values of two to three independent experiments each one in triplicate; ^b^ CC_50_: cytotoxic concentration (μM) to induce 50% death of non-infected cells, as evaluated with the MTT method in MT-4 cells; ^c^ EC_50_ (HIV-1, III_B_): effective concentration (μM) to inhibit by 50% HIV-1 (III_B_ strain) induced cell death, as evaluated with the MTT method in MT-4 cells; ^d^ SI: selectivity index calculated as CC_50_/EC_50_ ratio; ^e^ NVP: nevirapine.

**Table 6:** Anti-HIV-1 activity of PAS **59a**–**h** against the rRT.

Compound	IC_50_ ± SD (μM) ^a^		
	WT IIIB	Y181C	L100I
**59a**	0.45 ± 0.09	6.9 ± 2.3	7.4 ± 1.2
**59b**	0.40 ± 0.05	7.5 ± 1.4	8.5 ± 1.0
**59c**	0.40 ± 0.14	5.0 ± 1.5	10 ± 3.1
**59d**	0.27 ± 0.10	8.0 ± 2.0	14 ± 1.2
**59e**	0.90 ± 0.12	14 ± 2.5	>20
**59f**	>20	>20	>20
**59g**	>20	>20	>20
**59h**	>20	>20	>20
NVP	0.60 ± 0.1	>20	3.5 ± 0.18

^a^ Compound concentration required to inhibit the HIV rRT activity by 50%. SD: standard deviation.

**Table 7:**
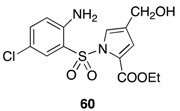
Anti-HIV-1 activity of PAS **60** in MT-4 cells and against rRT ^a^.

Compound	CC_50_^b^ (μM)	HIV-1 III_B_		IC_50_^e^ (μM)
		EC_50_^c^ (μM)	SI ^d^	
**60**	240	0.042	5333	0.05
NVP ^f^	>200	0.35	>571	0.64

^a^ Data are mean values of two to three independent experiments each one in triplicate; ^b^ CC_50_: cytotoxic concentration (μM) to induce 50% death of non-infected cells, as evaluated with the MTT method in MT-4 cells; ^c^ EC_50_ (HIV-1, III_B_): effective concentration (μM) to inhibit by 50% HIV-1 (III_B_ strain) induced cell death, as evaluated with the MTT method in MT-4 cells; ^d^ SI: selectivity index calculated as CC_50_/EC_50_ ratio; ^e^ Compound concentration required to inhibit the HIV rRT activity by 50%; ^f^ NVP: nevirapine.

## Dedication

Dedicated with respect and gratitude to Giorgio Stefancich, medicinal chemistry teacher.
